# Indicators of exposure to estrogenic compounds at Great Lakes Areas of Concern: species and site comparisons

**DOI:** 10.1007/s10661-018-6943-5

**Published:** 2018-09-06

**Authors:** Vicki S. Blazer, Heather L. Walsh, Cassidy H. Shaw, Luke R. Iwanowicz, Ryan P. Braham, Patricia M. Mazik

**Affiliations:** 1 U.S. Geological Survey, National Fish Health Research Laboratory, Leetown Science Center, 11649 Leetown Road, Kearneysville, WV USA; 2 College of Agriculture and Forestry, West Virginia University, Morgantown, WV 26506 USA; 3 Present Address: Vermont Agency of Natural Resources, Fish and Wildlife Department, UVM Hill Science Building, 105 Carrigan Drive, Burlington, VT 05405 USA; 4 U.S. Geological Survey, Cooperative Fish and Wildlife Research Unit, West Virginia University, Morgantown, WV 26506 USA

**Keywords:** Biomarkers, Estrogenic contaminants, Adverse effects, White sucker, Smallmouth bass, Largemouth bass

## Abstract

Adverse effects resulting from potential exposure of wild fishes to estrogenic endocrine disruptors were assessed at seven United States Great Lakes Areas of Concern using biomarkers ranging from organismal (gonadosomatic indices) to tissue/plasma (histology, plasma vitellogenin) and molecular (hepatic gene transcripts) levels. Biomonitoring was conducted on pelagic, top predator species, largemouth *Micropterus salmoides* and smallmouth *M. dolomieu* bass and benthic, omnivorous white sucker *Catostomus commersonii*. Seasonal (spring and fall) comparisons were conducted at select sites. Intersex (testicular oocytes), plasma vitellogenin, and hepatic vitellogenin transcripts were commonly observed in bass species. Testicular oocyte severity was positively, although weakly, correlated with plasma vitellogenin, hepatic transcripts of vitellogenin, estrogen receptor α, and estrogen receptor β2, while negatively correlated with androgen receptor β and phosphoenolpyruvate carboxykinase. No testicular oocytes were observed in white sucker; however, plasma vitellogenin and hepatic vitellogenin transcripts were commonly detected in the males. The results demonstrate the importance of utilizing multiple endpoints to assess exposure to estrogenic compounds as well as the importance of choosing sensitive species.

## Introduction

Great Lakes Areas of Concern (AOCs) are defined by the U.S.–Canada Great Lakes Water Quality Agreement, Annex 1 of the 2012 Protocol as “geographic areas designated by the Parties where significant impairment of beneficial uses has occurred as a result of human activity at the local level” (www.epa.gov/great-lakes-aocs). Within the AOC framework, 14 beneficial use impairments (BUIs) were defined that include a number which focus on the health of fish and wildlife, such as degraded fish and wildlife populations, fish tumors or other deformities, and bird or animal deformities or reproductive problems (www.eps.gov/great-lakes-aocs/beneficial-use-impairments). The Great Lakes Restoration Initiative (GLRI) is a multiagency effort that began in 2010 to “protect and restore the chemical, physical, and biological integrity of the Great Lakes Basin ecosystem.” One of the priorities of this initiative is reducing the release of toxic substances and accelerating remediation and restoration of historically contaminated sites such as AOCs (https://www.glri.us/action-plan).

Historically, the focus of remediation at AOCs has been on legacy contaminants such as metals, polycyclic aromatic hydrocarbons (PAHs), and polychlorinated biphenyls (PCBs). The “fish tumors or other deformities” BUI, for which PAHs have been identified as a key risk factor (reviewed by Rafferty et al. 2009), remains present (Blazer et al. 2014a and b, 2017), despite millions of dollars in clean-up and remediation, suggesting perhaps other risk factors need to be considered.

It has been recognized that chemicals of emerging concern (CECs) including substances such as biogenic hormones (human and animal), brominated flame retardants, pharmaceuticals, personal care products, plasticizers, current use pesticides, and detergents may contribute to impaired health of Great Lakes fish and wildlife. There has been a substantive increase in the detection of CECs within the Great Lakes watershed (Metcalfe et al. 2003; Wu et al. 2009; Li et al. 2010; Csiszar et al. 2011; Lee et al. 2012; Yang et al. 2012; Blair et al. 2013; Ferguson et al. 2013; Baldwin et al. 2016; Choy et al. 2017; Elliott et al. 2017), but fewer studies documenting adverse effects in aquatic biota, particularly at AOCs (Kavanagh et al. 2004; Mitchelmore and Rice, 2006; Klečka et al. 2010; Lozano et al. 2012; Simmons et al. 2014). Many CECs are not regulated by state or Federal water quality programs, contributing to the exposure of aquatic organisms to complex mixtures of legacy contaminants and CECs throughout their lives. Much of the emphasis on effects of CECs has been focused on reproduction, with the most documented effects being plasma or hepatic transcripts of vitellogenin in male or immature fishes and testicular oocytes (TO) or intersex (Sumpter and Jobling 1995; Abdel-moneim et al. 2015; Hiramatsu et al. 2006) as a result of exposure to estrogenic contaminants. However, these chemicals can also have adverse effects on the immune system (Milla et al. 2011; Casanova-Nakayama et al. 2011) and play a role in carcinogenesis (Birnbaum and Fenton 2003; Soto and Sonnenschein 2010).

The number of potentially harmful compounds, both legacy and CECs, as well as the spatial and temporal variation in concentrations make it difficult to assess risk based on chemical analyses of water/sediment or even fish tissue. Some chemicals accumulate in tissues while others can have significant effects at low environmental concentrations (picomolar or nanomolar) during key developmental periods, but not bioaccumulate. Additionally, we often have little knowledge of the cumulative effects (synergistic, additive, antagonist) of the complex mixtures present in most environments. These complex mixtures of chemicals may pose a substantial but poorly understood or documented threat to aquatic ecosystems, including tributaries of the Great Lakes. Consequently, there has been increased emphasis on development of effects-based tools to assess these cumulative impacts (Dubé and Munkittrick 2001; Connon et al. 2012). Effects-based tools or biomarkers can range from organismal to molecular level indicators (Huggett et al. 1992; Van Aggelen et al. 2010). The Great Lakes Chemicals of Emerging Concern Advisory Work Group (www.ijc.org/files/publications/C220.pdf) concluded that “effects-based monitoring in the context of multiple stressors should be developed and implemented to supplement the current chemical monitoring regimes.” To address this need, a multidisciplinary, integrated program that included chemical analyses, bioassays (in vitro assays of surface-water samples or extracts and in vivo bioassays using laboratory-reared model fish species exposed in situ short term), and biological endpoints in wild-caught fish was conceived (Ekman et al. 2014). A suite of biological endpoints to detect adverse effects in wild fishes was developed (Blazer et al. 2014c).

Species may differ in sensitivity due to genetic and physiological attributes, timing of spawning, or habitat usage. The fish species of choice for this study were white sucker *Catostomus commersonii* (benthic species) and largemouth bass *Micropterus salmoides* or smallmouth bass *M. dolomieu* (pelagic species). *Micropterus* species have been shown to be sensitive species for endocrine disruption studies, specifically responding to estrogenic compounds by induction of vitellogenin (Vtg) and TO in male bass (Blazer et al. 2014d, 2007; Hinck et al. 2009; Iwanowicz et al. 2009; Kellock et al. 2014; Yonkos et al. 2014; Iwanowicz et al. 2016; Abdel-moneim et al. 2017; Lee Pow et al. 2017). White sucker (WS) have also been used for monitoring effects of CECs on wild fish populations (Munkittrick et al. 1998; Doherty et al. 2003; Dorval et al. 2005; Woodling et al. 2006; Vajda et al. 2008) and are an indicator species for the fish tumor BUI at Great Lakes AOCs (Blazer et al. 2014a, 2017).

The experimental design of this project was a reconnaissance study that encompassed a broad geographical range focused on Great Lakes AOCs. Multiple biomarkers of exposure to estrogenic contaminants were assessed in wild fishes for site comparisons. Biomarker responses were also compared among species in order to better document relative sensitivities. Additionally, the utility of wild fish monitoring was compared to risk assessments based on monitoring of chemical concentrations and short-term in situ exposure of model species.

## Methods

### Field methods

**Fig. 1:**
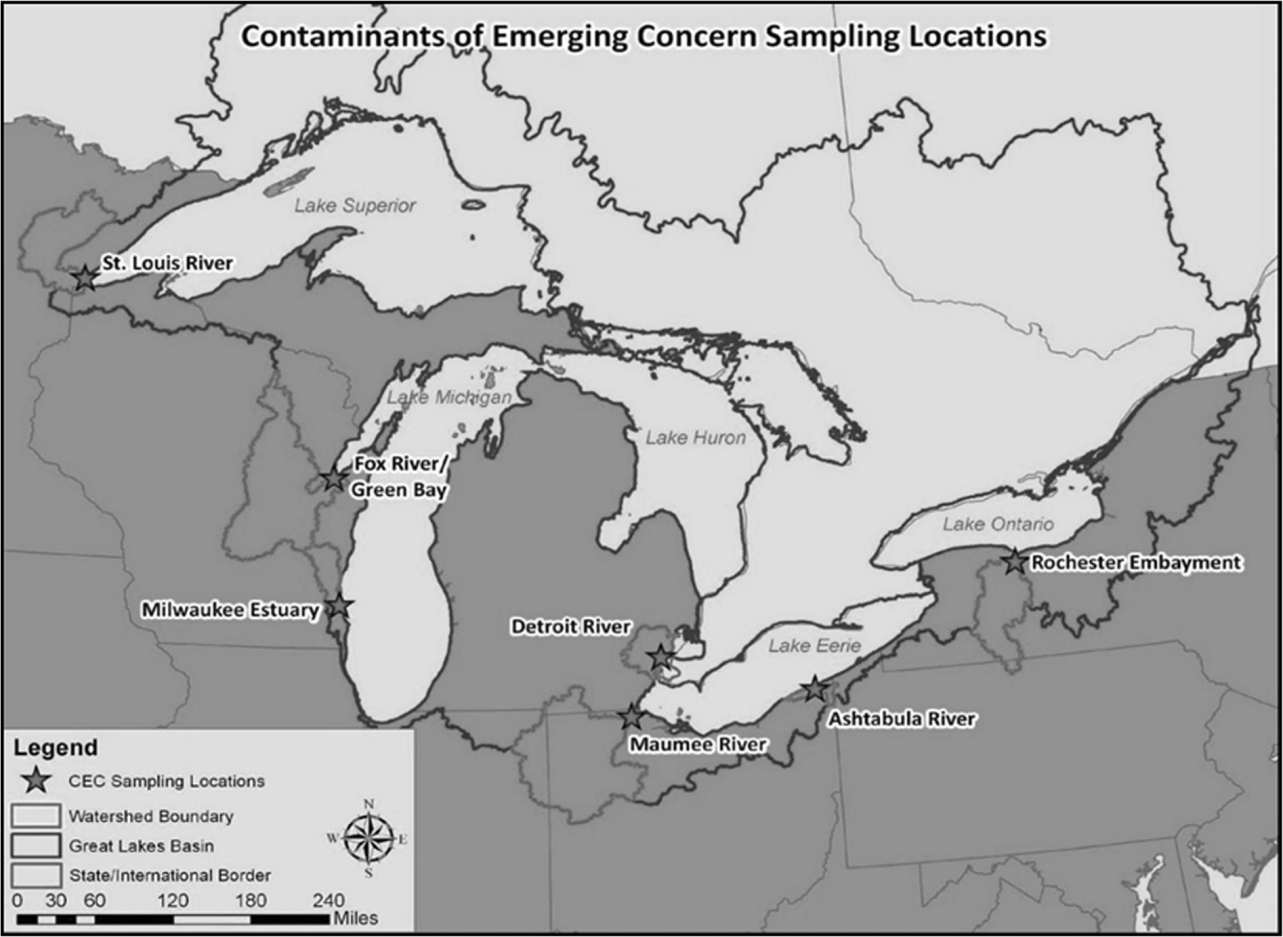
Map with location of the Areas of Concern sampled

Fish were collected from sites within seven AOCs (Fig. 1) in fall 2010 and spring 2011. Geographically, sites ranged from the Rochester Embayment AOC on Lake Ontario to the St. Louis River and Bay AOC on Lake Superior. The Rochester Embayment includes the entire Genesee River basin as well as the embayment formed by the indentation along the Monroe County shoreline. Fish were collected in the lower river portion of this AOC in both fall and spring. The St. Louis River becomes a freshwater estuary as it approaches Lake Superior and the AOC includes the lower river, estuary, and numerous bays. In the fall, fish were collected throughout the St. Louis Bay portion of the AOC. In the spring, one collection was made close to the Western Lake Superior Sanitary District wastewater treatment plant outfall (A) and one close to the mouth of Keene Creek and Minnesota Power’s Hibbard plant outfall (B). Two sites were located on Lake Michigan, the lower Milwaukee and Menomonee rivers in the Milwaukee Estuary AOC and the lower Fox River in the Lower Green Bay and Fox River AOC. Swan Creek, within the Maumee River AOC, and the lower Ashtabula River AOC are located on Lake Erie. The Detroit River AOC is a 44-km waterway between Lake St. Clair and Lake Erie. Additional site details are available in Choy et al. (2017).

Spring sampling targeted the prespawn period, while the fall sampling targeted the period of recrudescence. Monitoring was conducted at locations suspected of having biologically relevant concentrations of CECs and legacy contaminants, due to land use history, as well as known point and non-point sources. Fish sampling was done in conjunction with chemical analyses of discrete water and sediment samples (Lee et al. 2012; Choy et al. 2017). Attempts were made by personnel of the U.S. Fish and Wildlife Service to collect 20 mature fish (10 males and 10 females) each of two species at each site by boat electrofishing or fyke nets. Fish were euthanized with a lethal dose of Finquel (Argent Chemical Laboratories, Inc., Redmond, WA), weighed, and measured. A blood sample was taken from each fish using heparinized syringes. The blood was stored on wet ice until centrifuged (same day) and plasma aliquoted into two cryovials. Plasma samples were stored at − 80 °C until analyzed. A comprehensive necropsy-based assessment to document grossly visible abnormalities was completed on all fish collected. Gonads were removed and weighed to calculate the gonadosomatic index (GSI). In most cases, sex could be determined by gross examination of the gonads; however, it was verified microscopically. Gonadosomatic index (gonad weight/body weight × 100), an indicator of gonadal development or activity (Gross et al. 2002; Barrett and Munkittrick 2010), was calculated. One whole gonad or at least five sections if large, multiple pieces of liver and any observable skin abnormalities were placed in Z-fix (Anatech Ltd, Battle Creek, MI) for subsequent histological analyses. Two to three small pieces of liver (approximately 25 mg) were placed in RNAlater™ Stabilization solution (ThermoFisher, Waltham, MA) for gene transcript enumeration. Otoliths were removed for aging. More detail on field methods can be found in Blazer et al. (2014c).

### Laboratory methods—plasma vitellogenin (Vtg)

Plasma Vtg concentrations were quantified using a direct enzyme-linked immunosorbent assay (ELISA) as previously described (Blazer et al. 2014c). Primary antibodies used to detect Vtg were monoclonal antibodies purchased from Cayman Chemical (Ann Arbor, Michigan) and included ND-3G2 for SMB and LMB (Biosense Laboratories, Bergen, Norway). The Vtg standards used for the assay were purified from plasma of SMB induced with estradiol and prepared by the laboratory of Nancy Denslow, University of Florida, College of Veterinary Medicine, Gainesville, Florida. Plasma Vtg in WS was determined using a commercially available ELISA kit for carp (Cayman Chemical), according to the manufacturer’s protocols. Concentrations of the unknowns were interpolated from the standard curves and using Softmax® Pro v6.2.2 software (Molecular Devices, Sunnyvale, California). The limit of detection was 1 μg/ml.

### Microscopic pathology

Five to eight sections of preserved gonad samples were trimmed into cassettes, routinely processed and embedded into paraffin. Blocks were sectioned at 6 μm and routinely stained with hematoxylin and eosin (H&E). Sections of gonads were used to confirm sex, determine stage of development, TO prevalence and severity as previously described (Blazer et al. 2007), and identify other significant abnormalities. At least five cross-sections of a testis were each scored from 0 to 4 based on the number of oocytes observed per section and whether oocytes were single, clustered (three or more) or exhibited a zonal arrangement. Scores for the sections were averaged to determine a mean severity for each fish. Previous studies have suggested 0–15% prevalence of TO in bass may be considered a low or background level (Blazer et al. 2007). We considered 15–50% to be moderate and above 50% to be a high prevalence.

### Transcript abundance analysis

Transcriptome development and methods for transcript abundance are described by Hahn et al. (2016). Subsets of these genes, typically associated with exposure to EDCs, were analyzed for differences between sites in SMB, LMB, and WS. In SMB and LMB, six genes, androgen receptor β (*arβ*), choriogenin (*chg*), estrogen receptor α (*erα*), estrogen receptor β1 (*erβ1*), estrogen receptor β2 (*erβ2*), and *vtg*, were chosen for analysis. Six housekeeping genes in SMB, phosphoenolpyruvate carboxykinase (*pepck*), RNA binding motif protein X-linked 2 (*rbmx2*), ribosomal protein L8 (*rpl8*), tata box binding protein (*tbp*), eukaryotic translation initiation factor 3D (*etif3d*), and β-actin (*β-act*), and six in LMB, *etif3d*, β-*act*, *rbmx2*, elongation factor 1α (*ef1α*), *rpl8*, and hypoxanthine phosphoribosyltransferase 1 (*hprt1*), were used for normalization. In WS, four genes associated with reproduction *ar*, *erα*, *erβ*, and *vtg* and six housekeeping genes *rbmx2*, *mus81*, *etif3d*, *ef1α*, *rpl8*, and *pepck* were chosen for analyses. Custom CodeSets for each species were designed and synthesized by Nanostring Technologies (Seattle, WA) for nCounter® analysis. Transcript abundance analysis was conducted on prepared liver tissue at the University of Pittsburgh Genomics and Proteomics Core Laboratory (Pittsburgh, PA). The nSolver Analysis Software (v2.0) was used for quality control of gene expression data. Following Nanostring quality control protocols, normalization was performed using the geometric mean of reference (housekeeping) genes.

### Statistical analyses

Data were compared using R statistical package (R Core Development Team 2016) or GraphPad version 5 (GraphPad Software, Inc., La Jolla, CA). For three or more groups, the Kruskal–Wallis followed by Dunn’s multiple comparisons test was used. If only two groups were compared, the Mann–Whitney test was used. Transcript abundance data for genes associated with reproduction were assessed for correlations with other endpoints. Correlation analysis, using Spearman’s rank correlation coefficient, was first performed for species and sexes separately. In the case of bass and correlations of TO with other endpoints, LMB and SMB were combined for analysis due to the low sample size of males.

## Results

### Collection summary

Fish were collected in fall 2010 from 21 September to 19 October and in spring 2011 from 12 April to 2 May. Attempts were made to collect the fish species at the same sites in fall and spring to compare biological effects seasonally. This was not always possible and seasonal comparisons could only be made with WS at St. Louis Bay and Swan Creek; LMB at Genesee River and Detroit River; SMB at St. Louis Bay (low sample sizes) and the Fox River.

### Morphometric analyses

Length, weight, age, condition factor, and prevalence of grossly observed lesions for all fish collected in 2010 and 2011 are available in Blazer et al. (2014b). All fish collected were mature adults with the exception of WS collected from the Detroit River. These fish were all smaller and younger (2–3 years of age) than those collected at other sites which ranged in age from 3 to 16 years for WS, 4–14 years for SMB, and 4–12 years for LMB.

### Sex ratio

The low sample size for each sex did not allow for a robust assessment of sex ratios at individual sites; however, there were a few observations that are noteworthy. No SMB males were collected at the Fox River in the spring and 78% of males collected at that site in the fall were intersex. Only one male WS was collected in the fall from this site. At the Detroit site, all of the WS collected in fall were males.

### Indicators of exposure to estrogenic compounds in males

Indicators of exposure to estrogenic compounds, including the presence of TO, plasma Vtg, and hepatic *vtg* transcripts, were detected in male fishes from all sites (Table 1). While TO were observed in both bass species, they were not observed in any WS. The prevalence of TO in SMB ranged from 33% (St. Louis Bay spring) to 78% (Fox fall, Milwaukee spring). No TO were observed in LMB collected at Genesee in the fall or Ashtabula (spring). A low prevalence (10%) was noted at Genesee in the spring and a moderate prevalence (20–25%) at Swan Creek in the fall and Detroit in the spring. The severity of TO (rated 1–4) in intersex males was also lower in LMB when compared to SMB, except at St. Louis in the spring when only three male SMB were captured (Table 1).

**Table 1:** Percentage of male fishes with indicators of estrogenic exposure

Site	Sample size	Testicular oocytes % (severity)^a^	% with plasma vitellogenin^b^	% with hepatic vitellogenin^c^
Smallmouth bass				
St. Louis Bay fall	4	50 (1.2 ± 0.4)	100	75
Fox River fall	9	78 (2.1 ± 0.1)	100	86
St. Louis spring	3	33 (0.2 ± 0.0)	100	100
Milwaukee River spring	9	78 (0.8 ± 0.2)	100	100
Largemouth bass				
Genesee River fall	14	0	100	29
Detroit River fall	12	0	83	100
Swan Creek fall	14	21 (0.4 ± 0.1)	20	54
Genesee spring	10	10 (0.2 ± 0.0)	60	80
Detroit spring	8	25 (0.4 ± 0.2)	75	100
Ashtabula River spring	12	0	92	100
White sucker				
St. Louis Bay fall	6	0	50	83
Detroit River fall	10	0	100	90
Swan Creek fall	9	0	0	78
St. Louis Bay A spring	17	0	18	83
St. Louis Bay B spring	16	0	75	88
Swan Creek spring	10	0	0	100
Milwaukee River spring	6	0	83	100

^a^Percentage of male bass with testicular oocytes (mean severity ± SE for positive fish)

^b^Percentage of male fish with plasma vitellogenin concentrations above 10 μg/ml

^c^Percentage of male fish with hepatic vitellogenin transcripts abundance above 16

Baseline concentrations of plasma Vtg have not been experimentally determined for male bass or WS. Since 0.1 to 10 μg/ml have been considered baseline in other species (Hiramatsu et al. 2006), 10 μg/ml was conservatively used as a benchmark threshold. The prevalence of male SMB with plasma Vtg was higher overall when compared to the two other species. At all sites, 100% of male SMB had Vtg concentrations above 10 μg/ml. This was also observed in LMB captured from the Genesee River (fall) and WS from the Detroit River (fall). All other sites at which LMB were collected had some individuals with plasma Vtg greater than 10 μg/ml, ranging from 20% (Swan Creek fall) to 92% (Ashtabula River spring). No male WS collected at Swan Creek in either season had plasma Vtg concentrations above 10 μg/ml, while the prevalence of male WS with Vtg at other sites ranged from 18% (St. Louis Bay site A) to 83% at Milwaukee River (Table 1).

**Fig. 2:**
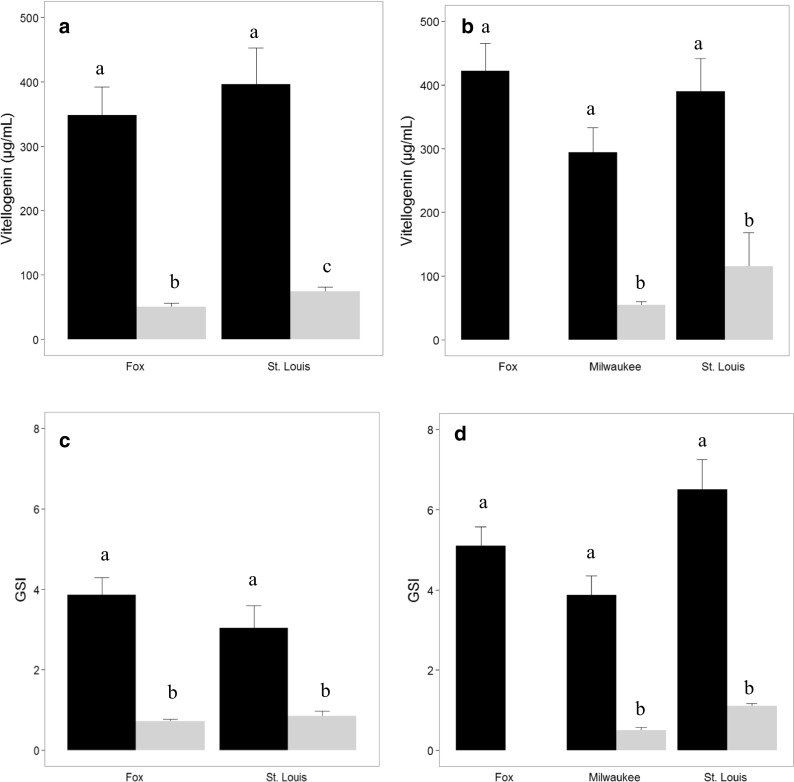
Plasma vitellogenin (μg/ml) concentrations and gonadosomatic index (GSI) of smallmouth bass collected at selected Great Lakes Areas of Concern. a Vitellogenin concentrations measured in the fall. **b** Vitellogenin concentrations measured in the spring. **c** GSI in the fall. **d** GSI in the spring. Black bars represent mean of female bass with standard error indicated; gray bars represent male bass. Bars with the same letter are not significantly different (*p* < 0.05)

Plasma Vtg concentrations varied among species, seasons, and sites. At all sites in both seasons, concentrations of Vtg in male SMB were significantly lower than those measured in females (Fig. 2a, b). There was a difference (*p* = 0.0205) between Vtg in male SMB from Fox River (50.7 ± 5.4 μg/ml) and St. Louis River (74.3 ± 6.7 μg/ml) in the fall (Fig. 2a); however, there was no difference (*p* = 0.6095) in GSI between the two sites (Fig. 2c). In the spring, no males were collected at the Fox River and there was no difference (*p* = 0.1025) in Vtg between SMB collected from the Milwaukee (54.4 ± 5.4 μg/ml) and St Louis rivers (115.6 ± 52.8 μg/ml) primarily due to the low sample size (4) at St. Louis and individual variability. The GSI was lower (*p* = 0.0161) in SMB collected from the Milwaukee River when compared to those from the St. Louis River (Fig. 2d).

**Fig. 3:**
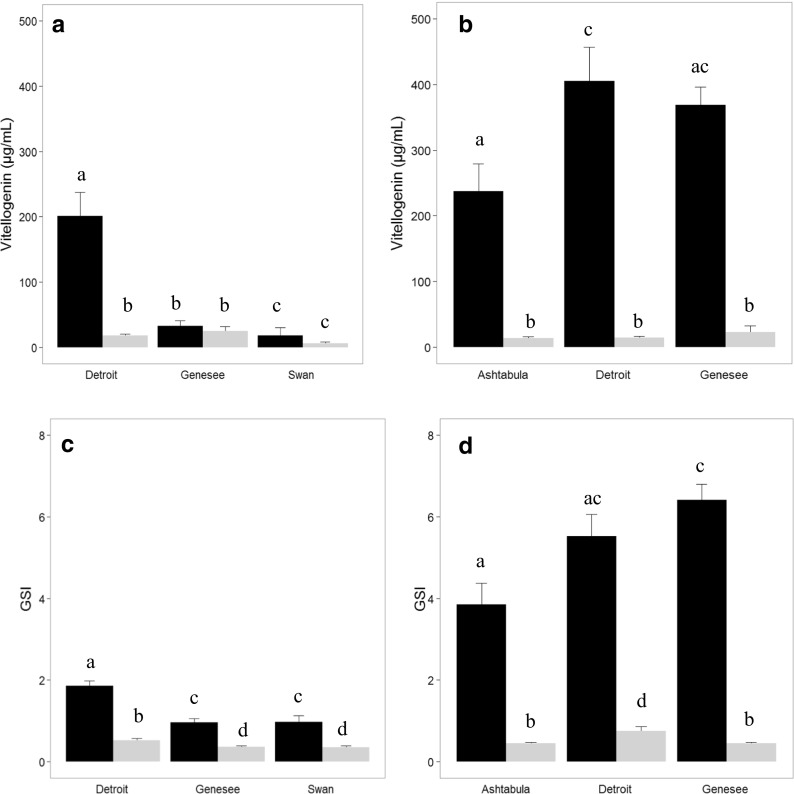
Plasma vitellogenin (μg/ml) concentrations and gonadosomatic index (GSI) of largemouth bass collected at selected Great Lakes Areas of Concern. **a** Vitellogenin concentrations measured in the fall. **b** Vitellogenin concentrations measured in the spring. **c** GSI in the fall. **d** GSI in the spring. Black bars represent mean of female bass with standard error indicated; gray bars represent male bass. Bars with the same letter are not significantly different (*p* < 0.05)

Mean plasma Vtg concentrations tended to be lower in male LMB than SMB in both seasons. There was a difference (*p* = 0.0004) among sites in the fall with LMB from Swan Creek having lower concentrations than all other sites (Fig. 3a). It is of note that a pairwise comparison of male and female LMB indicated a sex difference in Vtg concentrations at the Detroit River (*p* = 0.0011), but not at the Genesee River or Swan Creek in the fall. The GSI of Detroit LMB was higher (*p* = 0.0027) than at the other two sites (Fig. 3c). In the spring, females at all sites had significantly higher Vtg concentrations than males and there was no difference among sites for male Vtg concentrations (Fig. 3b), although GSI was higher at Detroit (*p* = 0.0005) than Genesee or Ashtabula rivers (Fig. 3d).

**Fig. 4:**
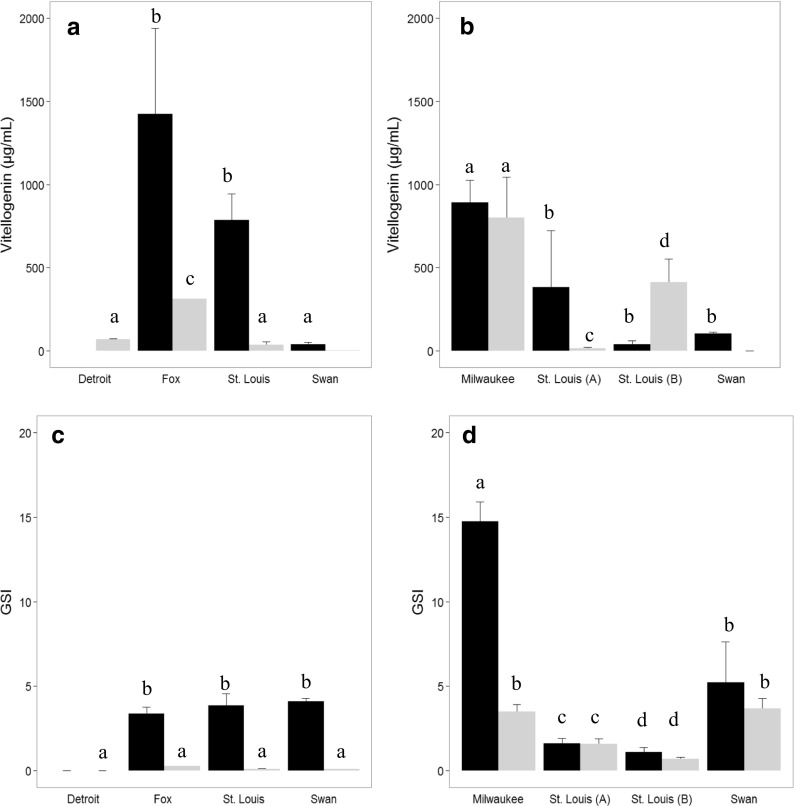
Plasma vitellogenin (μg/ml) concentrations and gonadosomatic index (GSI) of white sucker collected at selected Great Lakes Areas of Concern. **a** Vitellogenin concentrations measured in the fall. **b** Vitellogenin concentrations measured in the spring. **c** GSI in the fall. **d** GSI in the spring. Black bars represent mean of female suckers with standard error indicated; gray bars represent male suckers. Bars with the same letter are not significantly different (*p* < 0.05)

There was considerable variation among sites for mean Vtg concentrations in WS. Many of the male WS had either non-detectable Vtg or concentrations below 10 μg/ml. Plasma Vtg was below detection in all male WS collected in Swan Creek in the fall, while those collected at St. Louis (73.0 ± 1.0 μg/ml) and Detroit rivers (68.5 ± 3.6 μg/ml) had significantly higher (*p* = 0.006) concentrations, despite the Detroit males being immature. The one male collected from the Fox River had a high (312.7 μg/ml) concentration (Fig. 4a). There was no difference noted in GSI in the fall (Fig. 4b). In the spring, WS were collected at two different sites within St. Louis Bay. One site (A) was close to the Duluth Wastewater Treatment Plant, Duluth, MN, while the other site (B) was close to the Minnesota Power Plant. The overall mean concentration (both sites) of WS collected in St. Louis in the spring (83.9 ± 9.0 μg/ml) was similar to that observed in the fall (73.0 ± 1.0 μg/ml). However, male WS captured at site B actually had higher Vtg concentrations than the females (Fig. 4b). Another striking result for WS was seen at the Milwaukee site in the spring (Fig. 4b) where concentrations in male fish (963.3 ± 148.8 μg/ml) were similar (*p* = 0.6461) to those measured in female suckers (1041.2 ± 100.2). Mean WS Vtg for all sites sampled in the spring were different (*p* < 0.001) from each other. The GSI of WS from Swan Creek and Milwaukee were similar but different (*p* < 0.0001) from both St. Louis sites which were different from each other.

Although 100% of SMB had measurable plasma Vtg in the fall, only 75% at the St. Louis and 86% at the Fox had hepatic *vtg* transcripts. In the spring, 100% of SMB at St. Louis and Milwaukee had both plasma and hepatic Vtg (Table 1). At all sites and seasons except Genesee in the fall, a higher percentage of LMB had hepatic *vtg* than plasma Vtg. The male WS with hepatic *vtg* transcripts ranged from 78 to 100% and in all cases except Detroit in the fall, a higher percentage of suckers had hepatic *vtg* than plasma Vtg (Table 1).

Hepatic transcript abundance of select genes related to reproduction was measured. For most of the genes, large variations in transcript abundance were observed within sites and we did not statistically compare sites, although there were a number of notable observations. Although the sample size of male SMB was low at St. Louis in the spring, they exhibited high counts of both *vtg* and *chg*. It is also interesting to note that while *erα* transcripts were measured in male SMB at low abundance, most LMB did not have detectable levels. The exception was at Ashtabula (Table 2). Conversely, *erα* transcript abundance was considerably higher in WS males than in bass, particularly in the spring (Table 3). The *erα* to *erβ1*ratio of WS was 15.4 (Milwaukee River), 2.6 (Detroit River), and 5.2 (Swan Creek). In bass, it was considerably lower at 0.29 (Milwaukee River, SMB), 0.04 (Detroit River, LMB), and 0.02 (Swan Creek, LMB).

**Table 2:** Transcript abundance (median and (range)) in male bass from Great Lakes Areas of Concern

Sites and season	Vitellogenin	Estrogen receptor α	Estrogen receptor β1	Estrogen receptor β2	Choriogenin	Androgen receptor β	PEPCK^b^
Smallmouth bass							
St. Louis fall	219 (3–534)	12 (6–21)	74 (71–100)	304 (247–419)	198 (36–255)	416 (193–466)	1041 (684–2449)
Fox fall	793 (5–11,454)	10 (4–32)	115 (94–126)	376 (253–425)	154 (47–654)	186 (141–284)	2111 (1747–2247)
St. Louis spring	3549 (315–196,307)	30 (10–230)	123 (97–137)	408 (220–497)	4067 (758–18,491)	318 (237–343)	2467 (2401–2557)
Milwaukee spring	101 (14–275)	30 (13–48)	184 (135–205)	529 (373–942)	131 (84–2438)	389 (192–575)	2623 (1674–2857)
Largemouth bass							
Genesee fall	7 (1–54)	ND^a^	154 (74–202)	267 (97–566)	179 (16–602)	405 (314–628)	2862 (1378–5041)
Detroit fall	558 (17–4867)	ND	171 (23–252)	199 (13–267)	81 (15–644)	929 (46–1398)	2705 (179–3231)
Swan Creek fall	17 (5–7505)	ND	172 (138–265)	249 (75–315)	230 (96–1120)	384 (268–776)	2157 (655–3470)
Genesee spring	303 (1–7829)	ND	171 (114–217)	264 (50–496)	136 (54–905)	1171 (253–2937)	2761 (813–4598)
Detroit spring	154 (21–2825)	ND	145 (123–218)	191 (110–298)	409 (72–3006)	513 (344–747)	2556 (1749–5121)
Ashtabula spring	434 (84–9124)	13 (1–36)	197 (146–306)	330 (209–558)	343 (66–1536)	798 (334–1764)	3481 (2530–5016)

^a^None of the individuals had transcript counts above 16

^b^Phosphoenolpyruvate carboxykinase

**Table 3:** Transcript abundance (median and (range)) in white sucker collected at Great Lakes Areas of Concern

Sites and season	Vitellogenin	Estrogen receptor α	Estrogen receptor β	Androgen receptor β
White sucker males				
St Louis fall	71 (6–1390)	494 (275–775)	57 (4–115)	281 (207–358)
Swan Creek fall	181 (11–1507)	480 (4–960)	26 (14–164)	426 (356–823)
Detroit fall	152 (9–2177)	529 (205–938)	204 (171–392)	338 (288–522)
St. Louis A spring	27 (4–241)	1395 (559–2682)	309 (164–833)	541 (359–1497)
St. Louis B spring	83 (9–34,168)	812 (339–1801)	231 (83–498)	418 (179–596)
Swan Creek spring	64 (27–321)	1186 (384–2397)	288 (100–505)	530 (328–1112)
Milwaukee spring	226 (168–19,186)	1170 (950–1837)	88 (48–101)	372 (366–588)
White sucker females				
St Louis fall	250,831 (94–796,064)	1167 (285–2149)	69 (20–222)	331 (167–523)
Swan Creek fall	101,611 (2–247,197)	1002 (2–1806)	18 (12–200)	260 (175–564)
Fox fall	127,413 (36–591,506)	867 (235–1651)	21 (9–110)	203 (100–431)
St. Louis A spring	1411 (1266–3151)	884 (730–1162)	369 (278–488)	443 (425–470)
St. Louis B spring	29 (16–521,315)	1083 (519–1430)	401 (29–528)	374 (140–588)
Swan Creek spring	83,365 (14,396–1,459,791)	1396 (913–7810)	307 (69–438)	360 (266–549)
Milwaukee spring	609,398 (114,588–1,259,236)	2855 (1276–6165)	85 (6–220)	468 (241–884)

Correlation analyses were run with hepatic transcripts and TO, combining sites and seasons. Moderate positive correlations with *erα* and *erβ2* and negative correlations with *arβ* and *pepck* were observed (Table 4).

**Table 4:** Significant correlations of specific biomarkers

Parameter	Parameter	Spearman rho	*p* value
Male			
Bass (both species) testicular oocyte severity	Plasma vitellogenin	0.3397	0.0011
	Hepatic vitellogenin transcripts	0.2462	0.0193
	Estrogen receptor α	0.3866	0.0002
	Estrogen receptor β2	0.3416	0.0010
	Androgen receptor β	− 0.3256	0.0017
	Phosphoenolpyruvate carboxykinase	− 0.2370	0.0245
Female			
Smallmouth bass gonadosomatic index	Plasma vitellogenin	0.3791	0.0086
	Hepatic vitellogenin transcripts	0.4148	0.0034
Largemouth bass gonadosomatic index	Plasma vitellogenin	0.8059	< 0.0001
	Hepatic vitellogenin transcripts	0.9112	< 0.0001
White sucker gonadosomatic index	Plasma vitellogenin	0.4249	0.0006
	Hepatic vitellogenin transcripts	0.8706	< 0.0001
Smallmouth bass estrogen receptor α	Plasma vitellogenin	0.6592	0.0014
Largemouth bass estrogen receptor α	Plasma vitellogenin	0.6596	< 0.0001
White sucker estrogen receptor α	Plasma vitellogenin	0.3139	0.0115

### Biomarkers in female fishes

In SMB collected at St. Louis and Fox, there was little difference in plasma Vtg levels measured in females during fall and spring nor were there differences among sites in either season (Fig. 2a, b). While the GSI increased between fall and spring, there was no difference among the sites in either season (Fig. 2c, d).

In female LMB, there was a significant increase in both plasma Vtg and GSI between fall and spring. In fall (Fig. 3a), female Detroit bass had higher (*p* = 0.0034) mean Vtg concentrations (201.0 ± 36.6 μg/ml) than Swan (27.4 ± 16.4 μg/ml) or Genesee (32.6 ± 8.3 μg/ml). In spring, mean plasma Vtg concentrations in LMB from Detroit (405.4 ± 51.3 μg/ml) were higher (*p* = 0.0271) than those collected at Ashtabula (237.2 ± 42.0 μg/ml), while Genesee LMB (368.9 ± 27.3 μg/ml) were intermediate (Fig. 3b). The mean GSI of females at all sites was significantly higher in the spring than fall. Mean GSI (Fig. 3c) in the fall was highest at Detroit (1.85 ± 0.13) and lower (*p* = 0.0012) at Genesee (0.96 ± 0.10) and Swan (0.96 ± 0.16). The mean GSI of Genesee LMB in spring (6.4 ± 0.4) was higher (*p* = 0.0071) than at Ashtabula (3.8 ± 0.5) while Detroit WS (5.5 ± 0.5) were intermediate (Fig. 3d).

In female WS, the highest Vtg concentrations in fall were measured at the Fox (1424.1 ± 515.1 μg/ml) and St. Louis (785.4 ± 158.0 μg/ml) which were higher (*p* < 0.01) than the concentrations in suckers from Swan Creek (40.1 ± 12.1 μg/ml) and Detroit (69.0 ± 5.5 μg/ml). At Detroit, the Vtg concentrations of females were similar to those of males (Fig. 4a). Concentrations of Vtg in spring (Fig. 4b) at Swan Creek (103.2 ± 9.9 μg/ml) were similar to fall concentrations. Concentrations were higher (*p* = 0.0008) in WS from Milwaukee (1041.2 ± 100.1 μg/ml) than Swan, St. Louis A (382.6 ± 340.0 μg/ml), or St. Louis B (37.8 ± 22.4). Fall GSI of WS females were similar at all sites (Fig. 4c). In spring (Fig. 4d), GSI of WS females was higher (*p* = 0.0011) at Milwaukee (14.8 ± 1.1) than St. Louis A (3.8 ± 3.1) and St. Louis B (0.03 ± 0.01) but not Swan (5.2 ± 2.4). Transcript abundance in female bass (Table 5) varied greatly within and among sites.

**Table 5:** Transcript abundance (median and (range)) in female bass from Great Lakes Areas of Concern

Sites and season	Vitellogenin	Estrogen receptor α	Estrogen receptor β1	Estrogen receptor β2	Choriogenin	Androgen receptor β	PEPCK^a^
Smallmouth bass							
St. Louis fall	450,787 (291,785–490,069)	833 (620–833)	103 (99–112)	249 (242–256)	27,782 (23,548–27,808)	286 (180–386)	1150 (839–1917)
Fox fall	587,845 (14,138–769,339)	868 (83–1896)	145 (99–183)	236 (134–551)	31,725 (8025–39,500)	162 (121–327)	1665 (1093–2665)
St. Louis spring	570,017 (351,439–970,560)	2625 (2180–3092)	166 (130–235)	252 (247–255)	42,887 (28,453–69,395)	201 (53–297)	1792 (1661–1923)
Fox spring	537,953 (274–1,130,913)	1542 (14–3528)	173 (98–256)	327 (223–2005)	45,284 (78–75,800)	203 (62–469)	1317 (367–2575)
Milwaukee spring	292,314 (1063–479,371)	1315 (165–1556)	112 (72–194)	301 (161–471)	18,292 (2805–31,698)	129 (91–270)	1927 (1482–2685)
Largemouth bass							
Genesee fall	13,566 (21–38,143)	478 (142–882)	144 (100–215)	219 (142–466)	14,952 (3063–23,568)	378 (241–515)	2487 (1453–3393)
Detroit fall	319,197 (14,686–439,733)	1073 (137–1306)	127 (94–177)	143 (105–196)	27,561 (19,258–30,644)	249 (198–460)	2074 (941–2751)
Swan Creek fall	1903 (33–51,519)	63 (3–271)	170 (92–260)	124 (87–362)	4093 (246–25,416)	344 (220–620)	1654 (1082–1887)
Genesee spring	1,094,226 (916,353–1,314,136)	1979 (1464–3344)	129 (69–227)	260 (138–312)	59,494 (37,058–85,483)	153 (95–232)	1174 (922–1701)
Detroit spring	950,886 (184,113–1,341,129)	2160 (889–3859)	132 (64–233)	167 (76–277)	43,698 (10,624–62,679)	187 (80–400)	1746 (1009–2963)
Ashtabula spring	588,333 (284–948,095)	2216 (148–3034)	170 (106–267)	174 (140–211)	31, 696 (8484–47,085)	137 (115–250)	1972 (1542–2557)

^a^Phosphoenolpyruvate carboxykinase

We examined the association between GSI and Vtg in female fishes. While SMB and WS GSI were moderately correlated with plasma Vtg, there was a stronger association noted in LMB (Table 4). The GSI of all species was more strongly correlated with *vtg* transcripts than Vtg, especially in LMB and WS. Plasma Vtg was also correlated with transcript abundance of *erα* in all species (Table 4).

## Discussion

The reconnaissance of wild fish species at select Great Lakes AOCs indicate that fishes captured and presumably residing within these areas are exposed and responding to estrogenic EDCs. Indicators of exposure were observed at all the AOCs sampled, although there were differences among species and sites in the type and magnitude of response (Table 1). The findings emphasize the importance of utilizing multiple endpoints as well as more than one species. While SMB and LMB males with TO were observed at all sites, except Ashtabula, no TO were observed in WS from any of the sites. However, testicular tumors were observed in WS at the Milwaukee site (Blazer et al. 2017). All species showed induction of Vtg in males at all sites, either through measurement of plasma or gene transcripts. While induction of Vtg and TO are both widely used as indicators of exposure to estrogenic compounds, each endpoint can be indicative of different exposure time periods. Laboratory exposure studies indicated that although TO can be induced in adult and juvenile fishes, early life stages are the most sensitive (Van Aerle et al. 2002; Liney et al. 2005). Plasma Vtg is detectable within days of exposure to estrogens and may persist in circulation for months, while hepatic *vtg* transcripts may be detected in hours and turnover is generally more rapid (Hiramatsu et al. 2006).

The results presented also emphasize the importance of including wild fish monitoring to comprehensively assess the effects of complex mixtures and ecosystem health. When compared to chemical monitoring (Lee et al. 2012; Choy et al. 2017), cell-based bioassays and 4-day in situ exposure of fathead minnow *Pimephales promelas* (Li et al. 2017) or computed estradiol equivalency quotients (EEQ) for risk of endocrine disruption (Baldwin et al. 2016) monitoring of wild fish health revealed contaminant effects that would otherwise have remained undetected. Organic waste compounds were sampled at 57 Great Lakes sites between 2010 and 2013 and included five of the current sites: St. Louis, Milwaukee, Maumee (Swan Creek), Fox, and Genesee rivers. The chemical findings were used to calculate EEQ and the potential for induction of TO or Vtg. Four of the five sites included in the current study had zero potential for either TO or Vtg induction based on the calculated EEQ, with only the St. Louis River having a high potential for intersex induction and a medium potential of Vtg induction (Baldwin et al. 2016). Yet, at the Fox River, Maumee (Swan Creek), and Milwaukee River (Table 1), Vtg induction and TO prevalence were observed at high (> 50%) or medium (20–50%) levels in the wild fish sampled at these sites. This discrepancy between the actual observed response and the computed potential based on chemical detections is most likely due to species differences in sensitivity to various chemical (or chemical mixtures) exposure and to the fact that many chemicals such as phytoestrogens (Wang et al. 2016) and metformin (Niemuth et al. 2015), that may contribute to TO and Vtg induction, were not targeted by the analytical chemistry.

Species differences and/or exposure during sensitive time periods may also explain the differences in results between wild fishes and caged model fish species. At two of the AOC (Fox and Milwaukee), caged fathead minnows were deployed at four sites for 4 days. Cell-based bioassays were also used to determine estrogenic and androgenic activity in water samples. Estrogenic activity was identified at one site in the Fox and androgenic activity at another site in the Fox; no other sites exhibited significant activity. There was no evidence for endocrine disruption in the fathead minnows based on ex vivo steroid production, plasma steroid concentrations, or *vtg* expression in males (Li et al. 2017). These results differ from those observed in the wild fishes at the same site. However, the benefit of the cell-based assays and caged fathead minnows is the ability to identify local differences within an area and focus on proximity to specific point sources. Hence, these methods can be powerful when integrated with wild fish sampling.

Species differences in response to environmental and experimental exposures to estrogenic contaminants have been observed in numerous studies (Kavanagh et al. 2004; Routledge et al. 1998; Van den Belt et al. 2003; Tyler et al. 2005; Palace et al. 2009; Lange et al. 2012). A previous study in the Chesapeake Bay drainage found that WS and SMB collected at the same sites both had measurable plasma Vtg; however, only the SMB had TO (Blazer et al. 2014d). In wild fish exposed to ethynylestradiol added to a whole lake, TO were also not observed in WS but were in pearl dace *Margariscus margarita* and fathead minnow (Palace et al. 2009). These differences may be a result of different habitat usage (particularly during early life stages), differential chemical uptake, metabolism and bioconcentration, as well as physiological differences including metabolic rate, enzyme activities, and hormone receptors.

The current study was not designed to identify cause(s) of the observed effects; however, it was performed in conjunction with water contaminant analyses. Multiple water samples were taken throughout the sampled AOCs (Lee et al. 2012). At most sites, water samples were collected within 2 days of the fish sampling. At the St. Louis site in the spring, water samples were collected months after the fish sampling and therefore are not included. A large number of CECs were targeted including 9 alkylphenols, 8 flavors/fragrances, 17 hormones, 9 PAHs, 11 pesticides, 51 pharmaceuticals, 9 plasticizers/flame retardants, 4 sterols, and 16 others (Choy et al. 2017). Concentrations of most chemicals varied throughout a particular site with many below detection. For these reasons, the highest concentration measured within an individual AOC was compared with the biological endpoints measured in the wild fishes. There were few detects of hormones and other compounds that have been associated with reproductive endocrine disruption (Table 6). Detroit was the only site at which androgens were measured in the spring and had the highest concentration in the fall (Table 6). It is interesting that no female WS were collected from that site. However, this could be coincidental and more focused research and monitoring is necessary.

**Table 6:** Concentrations (ng/L) of chemicals in water samples from sites within selected Areas of Concern

Site	Estrone	Estriol	17β-Estradiol	4-Androstene 3,17-dione	*Cis*-androsterone	Dihydrotestosterone	Bisphenol A	Atrazine	Metolachlor	Nonylphenols^a^	Octylphenols^b^
Fall											
St. Louis	5.6	BD^c^	BD	BD	BD	BD	328	BD	BD	3162	1087
Detroit	0.9	BD	BD	0.9	1.3	BD	BD	26	BD	BD	BD
Genesee	0.4	BD	BD	BD	BD	BD	BD	35	11	BD	BD
Swan Creek	0.9	BD	BD	BD	0.7	BD	72	23	BD	BD	14
Fox	BD	BD	BD	BD	BD	BD	83	117	21	BD	BD
Spring											
Detroit	2.5	1.6	2.2	3.6	8.5	3.4	90	42	12	2631	209
Genesee	BD	BD	BD	BD	BD	BD	57	26	21	BD	53
Swan Creek	0.5	BD	BD	BD	BD	BD	63	83	95	778	BD
Fox	1.0	BD	0.2	BD	BD	BD	67	594	18	539	99
Ashtabula	BD	BD	BD	BD	BD	BD	BD	22	11	BD	BD
Milwaukee	1.2	BD	BD	BD	BD	BD	92	77	42	1576	161

^a^Sum of all isomers of 4-nonylphenol, 4-nonylphenol monorthoxylate, and 4-nonylphenol diethoxylate

^b^Sum of 4-*n*-octylphenol, 4-*tert*-octylphenol, 4-*tert*-ocylphenol monoethoxylate, and 4-*tert*-ocylphenol diethoxylate

^c^*BD* below detection

Estrone was the only steroidal estrogen detected in the fall and ranged from below detection at the Fox River to 5.6 ng/l at the St. Louis River site. In the spring, additional estrogens were occasionally detected and totals of steroidal estrogens were highest at Detroit River (6.3 ng/l), moderate at Swan Creek (0.5 ng/l), Fox (1.2 ng/l), and Milwaukee rivers (1.2 ng/l), and below detection at Genesee and Ashtabula rivers. The below detections at Ashtabula and Genesee are consistent with the low levels of TO at these sites, and the higher prevalence at the Fox in the fall is consistent with the higher steroidal estrogen concentrations. However, Milwaukee also had a high prevalence of TO but moderate concentrations of detected estrogens. St. Louis, Detroit, and Milwaukee (all with TO in bass) also had relatively high concentrations of nonylphenol and octylphenols (Table 6). These synthetic chemicals are generally considered weak estrogens, at least in model fish species. However, species responses to various exposure mixtures may differ. Another centrarchid, bluegill *Lepomis macrochirus*, showed a higher sensitivity for transactivation of *erα* when exposed to nonylphenol, octylphenol, bisphenol A, and DDT metabolites than fathead minnows (Miyagawa et al. 2014). Recently, it was shown that *erα* and *erβ* isoforms cloned from fathead minnow and bluegill responded differently to agricultural mixtures (eight contaminants) versus urban (11 contaminants) mixtures. Bluegill were the most sensitive to both mixtures showing a dose response in induction of *erα*, while the agricultural mixture only induced *erβ* at the highest concentration and there was only low induction of *erβ* by the urban mixture. Conversely, fathead minnow *erα* was only induced by the highest concentration of the agricultural mixture and the highest two concentrations of the urban mixture. However, the fathead minnow *erβ* was more responsive to the urban mixture than the bluegill *erβ* (Kohno et al. 2017). Interestingly, a survey of centrarchids at 20 riverine sites in North Carolina showed a distinct difference between *Lepomis* and *Micropterus*. A higher incidence and severity of TO was observed in the bass (*Micropterus*) species when compared to *Lepomis* (Lee Pow et al. 2017).

The differences among estrogen receptor responsiveness may not only explain the species differences observed in this study but also the extent and magnitude of the responses observed in relation to previous studies with bass species. The AOCs sampled here are in close proximity to major urban areas. In general, the prevalence and severity of TO were lower than what has been reported in bass from agriculturally impacted sites, where it is not uncommon to have 80–100% prevalence (Iwanowicz et al. 2009; Blazer et al. 2012, 2014d). These areas have also experienced bass mortality and population declines (Blazer et al. 2010; Smith et al. 2015). To our knowledge, effects such as these have not been noted at AOCs. In fact, Fayram et al. (2014) found SMB catch per effort values were not significantly different than least impacted rivers with suitable habitat. Based on relative *er* abundance and responsiveness, it may be that bass are more sensitive to the complex mixtures in agriculturally dominated watersheds.

The hepatic gene expression also provides additional information on potential adverse effects pathways occurring as a result of exposure to estrogenic chemicals in these species. A few previous studies have found a relationship between TO development and either plasma Vtg or hepatic *vtg* (Kidd et al. 2007; Iwanowicz et al. 2016; Abdel-moneim et al. 2017). To our knowledge, positive correlations of TO presence/severity with *erα* and *erβ2* and negative correlations with *arβ* or *pepck* have not been previously observed in wild fishes. This suggests that both estrogenic (leading to increased estrogen receptors) and anti-androgenic (leading to reduced androgen receptors) compounds may be involved in the induction of TO observed in bass. A previous study of estrogen receptor isotypes in LMB showed *erα* was highly expressed in the spring when plasma estradiol and Vtg were elevated, while *erγ* (*erβ2*) was only slightly up-regulated and *erβ* unchanged. Injection of males with β-estradiol induced *erα* but not *erβ* and *erγ* (*erβ2*) was moderately up-regulated (Sabo-Attwood et al. 2004). In our study, all three receptors were measured in male SMB in both seasons, although transcripts of *erβ2* were the most abundant. In male LMB *erα* was not expressed, while the other two receptors were moderately abundant in both seasons (Table 2). Given that both *erα* and *vtg* are induced by estrogens, this may suggest differences in activation thresholds between these closely related species. However, the species were collected at different sites, so other explanations are possible as well. Larger sample sizes at individual sites, as well as temporal sampling and more in-depth chemical analyses would be needed to better understand these differences.

The negative association of bass TO with *pepck* (significant but only moderately strong) is interesting in light of recent reports that exposure of fathead minnow fry to metformin induced TO (Niemuth et al. 2015), while exposure of adults induced *vtg* mRNA (Niemuth and Klaper 2015). Metformin, an anti-diabetic drug, is one of the most common pharmaceuticals entering the aquatic environment (Oosterhuis et al. 2013) although unfortunately not one of the chemicals analyzed at the sites in the current study. Inhibition of *pepck* expression in zebrafish has been suggested as a marker for exposure to anti-diabetic compounds (Elo et al. 2007). Concurrent monitoring for the presence of metformin in aquatic ecosystems and change in the abundance of the *pepck* biomarker in bass species is needed to better understand the impact on reproduction.

The majority of endocrine-associated effects monitored in wild fishes have focused on estrogenic chemicals and adverse effects on male fishes. However, there are studies that suggest effects in female fishes occur and may be more detrimental at the population level (Miller et al. 2007; White et al. 2017). In the current study, the correlations of GSI with plasma and hepatic vitellogenin suggest effects on females may be minimal, particularly in bass. In WS females, there was a strong correlation between *vtg* and GSI, but the relationship with plasma Vtg and GSI was weaker (Table 4). Unfortunately, one of the difficulties in assessing wild fishes, particularly when attempting to compare species and sites that are geographically separated, is fish may be at different stages of their reproductive cycle. Indeed, some of the fish we sampled in the spring were post spawn, although attempts were made to collect fish prespawn. Barrett and Munkittrick (2010), after a review of the characteristics of more than 60 fishes that have been used for reproductive studies in Canada, suggested the maximum sensitivity and best time to sample was 2 to 4 weeks prior to spawning for bass, but late fall for WS. All three species used are spring spawners, although WS spawn earlier than bass. However, even in the fall, there were discrepancies between the various endpoints in WS. For instance, GSI were similar at all three sites while Vtg was high at the Fox and St. Louis but low at Swan (Fig. 4). Further sampling, focusing on key periods for WS, would be needed to assess adverse effects on WS populations.

In conclusion, the suite of biological endpoints assessed in wild fish species indicates there is evidence of exposure to estrogenic chemicals at all of the AOC assessed. Adverse effects such as TO and Vtg induction in males were observed and were not predicted based on the chemical concentrations and caged fish studies. The results indicate bass species are more affected by estrogenic exposures when compared to WS, at least in terms of structural (TO induction) changes. The site differences will allow for prioritization of more comprehensive studies, including not only reproductive endocrine disruption but other adverse effects such as genotoxic/carcinogenic responses and responses to infectious disease.

“All applicable international, national guidelines for the care and use of animals were followed. Fish handling was conducted in accordance with the Leetown Science Center’s Institutional Animal Care and Use guidelines.”
